# Identification of key films and personalities in the history of cinema from a Western perspective

**DOI:** 10.1007/s41109-018-0105-0

**Published:** 2018-11-30

**Authors:** Livio Bioglio, Ruggero G. Pensa

**Affiliations:** University of Turin - Dept. of Computer Science, C.so Svizzera, 185, Turin, I-10149 Italy

**Keywords:** Complex networks, Network analysis, Citation analysis, Centrality, Cinema, Directors, Actors, Actresses

## Abstract

The success of a film is usually measured through its box-office revenue or through the opinion of professional critics; such measures, however, may be influenced by external factors, such as advertisement or trends, and are not able to capture the impact of a film over time. Thanks to the recent availability of data on references among movies, some researchers have started to use citations patterns as an alternative method for ranking movies. In this paper, we propose a novel ranking method for films based on the network of references among movies, calculated by combining four well known centrality indexes: in-degree, closeness, harmonic and PageRank. Our objective is to measure the success of a movie by accounting how much it has influenced other movies produced after its release, from both the artistic and the economic point of view. We apply our method on a subset of the IMDb (Internet Movie Database) citation network consisting of around 47,000 international movies, and we derive a list of films that can be considered milestones in the history of cinema. For each movie we also collect data on its year of release, genres and countries of production, to analyze trends and patterns in the film industry according to such features. We also collect data on 20,000 directors and almost 400,000 performers (actors and actresses), and we use the network of references and our score of movies for evaluating their career, and for ranking them. Since the IMDb dataset we employ is highly biased toward European and North American movies and personalities, our findings can be considered relevant principally for Western culture.

## Introduction

Cinema is characterized by a double-sided nature. From one side, filmmaking plays a leading role from a cultural point of view: universities all over the world offer majors courses on its history, language and techniques, while experts in the field have recognized it as the “seventh Art form”, at the same level of other classical creative expressions such as painting, music or poetry (Sadoul 1976). On the other side, filmmaking is now a profitable industry: it has been identified as one of the most valuable economic resources for a country (Bakker 2005), several companies support themselves with the production of movies, while people involved in the cinema industry (directors, performers, musicians, special effects technicians) can become celebrities known worldwide for their performances. Cinema has also a recognized impact on other industrial and cultural sectors as well: for instance, its influence on tourists’ choices (Bolan and Williams 2008) has fostered sponsorship of movies from tourism promotion associations. Therefore, a movie can be seen both as a form of art and as a product to sell; because of this dual, and in many cases divergent, perception of cinema, the success and importance of a movie is usually determined by either commercial or artistic criteria. Economical evaluations are based on the so-called box office, that is the amount of money raised from tickets sold in theaters and in the home video market (sales and rentals). Such measure has the advantage to be objective: best movies are simply the ones that sell more, like any other product. However, it has been observed that the financial performance of a film is related not only to its quality, but also to other factors, such as advertising, marketing expenditures, production costs, presence of movie stars in the cast (Prag and Casavant 1994), trends driven by word of mouth (Hennig-Thurau et al. 2015; Liu 2006), or to be part of a bigger franchise or the sequel of a successful movie (Dhar et al. 2012). In addition, such approach makes it difficult to compare movies of different ages, even when box office revenues are adjusted for inflation^1^, because these values may be inaccurate (Anderson et al. 2003), and sales may be influenced by economic performances and price level of the period when the movie has been released (Pautz 2002). Finally, the recent diffusion of video-on-demand technologies is changing the distribution of movies (Wasko 2011; Zhu 2001), making the release on theaters less important for its success. On the other hand, artistic judgments are purely subjective, since they are delivered by professional critics that evaluate a movie from the aesthetic and technical points of view. Film critics are persons with recognized expertise in the field of cinema, but, as any other human being, they may be influenced by trends of the moment or ideologies. In addition, critics tends to focus their attention on the artistic merits of a film, acclaiming movies with distinctive aesthetic and high intellectual level, that result difficult to be appreciated by the majority of viewers, usually more interested in the entertainment aspect of cinema (Holbrook 2005).

Summarizing, both common methods used for evaluating the success of films exhibit great limitations: for this reason, some researches have started to propose alternative techniques to tackle this problem. The most promising ones are based on the references that a movie receives from other ones released after it (Wasserman et al. 2015; Spitz and Horvát 2014; Wasserman et al. 2018; Canet et al. 2016): the network of citations among movies is collected, and a success score for each movie is computed through graph centrality algorithms, or other techniques borrowed from Network Analysis. The key intuition behind these methods is that a successful movie — it does not matter whether for economic or artistic merits — will be probably known and referenced by some of the successive ones, for honoring it or for trying to reproduce its outstanding performance. Such analyses are made possible thanks to the availability of data about the citations patterns among movies: the best-known public platform for collecting such (and other) data on movies is IMDb (Internet Movie DataBase), an online database storing several information about films and tv shows.

In this paper, we firstly introduce a score that estimates the importance of movies in the history of cinema, then we use it to evaluate the career of directors, actors and actresses, by considering their participation in top-scoring movies. Our success score is based on the network of references among movies, and aims at identifying those films that have had a central influence in the field of cinema, and can be considered a source of inspiration for many others. It is composed by a combination of four well-established centrality scores for graphs: in-degree, closeness, harmonic and PageRank. Other researchers have already employed similar techniques (such as Wasserman et al. (2015); Spitz and Horvát (2014); Canet et al. (2016)), but they selected different centrality scores for their measures: in “Motivations” section we explain in details the motivations under our choice of these four specific centralities. The network of references we use for calculating our score has been inferred from the publicly available subset of IMDb: it is composed by more than 47,000 films produced in several countries from 1920 to 2010. We apply our technique on this network to compute a score of importance for each movie in dataset: this score is then used to obtain a global ranking of the most significant movies. It is worth noting that, since the dataset employed in this study is highly biased toward films produced in Western countries (European and North-American ones), our results can be considered relevant preeminently for Western culture. For each movie, we also collect data on its year of first release, genres and countries of production, and analyze the patterns that emerge in film industry according to these features.

Finally, we use the score of movies also for evaluating the career of key personalities in cinema industry, i.e., directors, actors and actresses. Our technique ranks personalities according to the number of appearances (for actors and actresses) and film directions (for filmmakers) in top-ranked movies (based on our score) she has collected during her career. This method is based on the assumption that the success of a movie is partially due to her participation, or that the good performance of a film had helped to boost her career. As for movies, the score calculated for each person is used to reveal a global ranking of the most significant personalities in the history of (Western) cinema; we also study other secondary rankings focused on films features that show interesting behaviors.

The remainder of the paper is organized as follows: “Related works” section presents some related work; in “Analysis of the dataset” section we introduce the data used in our analysis and report some statistics about them; “Ranking method” section introduces our ranking method, and explains why we have chosen those particular centrality scores; we report the results of our analysis on films in “Results of ranking” section and those concerning the study of directors and performers’ careers in “Analysis of careers of directors, actors and actresses” section; finally, “Conclusions” section provides some concluding remarks.

## Related works

Network Science tools and techniques are widely used for quantifying the impact of works and individuals in a field. Significant resources for this task are represented by the networks of references between works and collaborations among personalities: for this reason, the fields where such features can be easily extracted have been extensively explored by both researchers and practitioners. In fact, thanks to the huge amount of publicly available data on citations between scientific papers, the majority of studies on this task concern scientific publications: such networks have been studied to analyze their structures (Sinatra et al. 2015), to find innovation trends (Renoust et al. 2016; Bioglio et al. 2017), and to quantify the impact of papers and authors (Kaur et al. 2015; Petersen et al. 2014). The most known outcomes of this kind of research are the measures proposed to estimate the scientific production, such as the highly diffused (and discussed) Hirsch’s h-index (Hirsch 2005).

### Collaboration and citation networks in the arts domain

In the artistic domain it is more difficult to construct a network of citations between individuals or works, because, contrary to scientific papers, influences are not explicitly reported in artistic products, as they must be inferred by human experts. For this reason, most research efforts have focused on analyzing collaboration networks instead of citation ones, in particular in the domains of music (Gleiser and Danon 2003; Park et al. 2007), where collaborations are made explicit on records, and cinema (Barabási and Albert 1999; Eom et al. 2008; Gallos et al. 2013), where collaborations can be easily inferred from movie credits. Nonetheless, some tentative has been done: in Elgammal and Saleh (2015), the authors construct a creativity implication network of the visual art domain, using a computer vision algorithm produced for quantifying similarity between artworks. For cinema, the availability of the references network produced by IMDb users has fostered many researches on this topic, in particular with the aim of inferring the importance of a movie in the history of this art domain. In Wasserman et al. (2015), the authors study the correlation between several metrics on movies (metacritic score^2^, IMDb rating, box office, number of citations received, and PageRank score) and the presence of movies in the United States Library of Congress’s National Film Registry (NFR), the selection of films for preservation in the Library of Congress of United States, but their analysis are limited to movies produced in United States. In Canet et al. (2016), the network of citations across movies supplied by IMDb is employed for studying the most inspiring movies and how their influence has evolved over the years. The authors found that the inspiration of recent movies comes predominantly from the ones produced in the 70’s and 80’s, with some films from classical periods that still have a huge influence. However, their ranking of the most influential movies is rather straightforward, being simply the number of citations received.

### Analysis of career and success

Many researches that propose measures to quantify the influence of works and individuals base their analysis on the number of references only: the more incoming citations a work (or a scientist) receives, the more impactful it is (Garfield 1955). In Sinatra et al. (2016), the authors use citation metrics for analyzing the career of award winning scientists, finding that highest-impact works in scientists’ career are randomly distributed within their entire career, i.e. the highest-impact papers of researchers can be published, with the same probability, in any moment of their research life.

Despite the fact that there are several research efforts aimed at determining the most important works or personalities in sciences, few efforts have been done for extending the studies to other domains, probably because of the lack of data. As regards the arts domain, in literature we can found only few applications of network science for evaluating the career of artists: in Vedres (2017), data on collaborations between jazz musicians on records are employed for predicting innovation and success, while in the field of Cinema the career of actors and actresses have been only studied for understanding their impact in the success of a film (Wallace et al. 1993). Other studies have addressed the problem of gender inequality in the career of actors and actresses (Simonton 2004; Taylor et al. 2012). In recent times, some researchers began to use metrics defined in a more complex way than the simple number of incoming arcs for determining the importance of nodes in a network of references. The authors of Spitz and Horvát (2014), for instance, use data made available by IMDb, in particular the network of references, for calculating an influence score for movies by means of a combination of several centrality metrics for graphs. Such metrics however highly takes into consideration the temporal distance between citations, emphasizing the impact of ancient movies on modern ones. In Zakhlebin and Horvát (2018), the authors investigate how top ranked items obtained from several centrality indexes may differ from expert opinions and popularity in different domains, findings substantial differences between the areas in terms of predictability of success as well as in determining which index is the best predictor.

### Relations and differences with authors’ previous work

The present paper is an extension of the analysis we proposed in Bioglio and Pensa (2018). In our previous paper, we presented our method for ranking movies and we applied it on IMDb dataset, discussing the relevance of movies in the global ranking; we also studied the ranking of movies labeled with certain tags, such as a genre or a country of production, interpreting the result. Here we briefly present the results of the same method, but applied on the IMDb dataset purged of movies labeled as “adult” or “short”, because the former is weakly related to the other genres (with its own industry and “appreciators”), and we focus on full-length and medium-length movies only. In addition, here we propose a different analysis of the tags associated to movies and, above all, we introduce a new technique for evaluating the career of personalities involved in film production: such technique is strongly linked to the rank of movies obtained from our score, because it considers only persons involved in top-ranked productions.

## Analysis of the dataset

IMDb is an online database of movies and TV series, featuring metadata such as year, country, genre, cast, production crew, budget, box office revenue, and so on. As of April 2018, IMDb includes information on more than 4 million titles (including episodes of TV series) and 8 million personalities (cast or crew)^3^. The site allows users to register and to expand the database in a collaborative way, by submitting new material, editing existing information and rating movies stored in the database.

Registered users can also record references between entries, choosing among several kinds of relationships, from remakes to acknowledged source of inspiration: such data on connections between movies are the core of our method.

For our analyses, we use data extracted from a subset of the entire IMDb database, made available by the website for research and non-commercial use^4^. For each movie, we collect its title, year of release, countries of production, genres, references, directors and crew (only actors and actresses), removing all movies not involved in a reference connection, i.e. that neither references nor are referenced, and all movies labeled with genres “short” (to study only full-length and medium-length films) and “adult”: this filtering procedure is the main difference on the ranking method with respect to the dataset employed in our previous analysis (Bioglio and Pensa 2018). Some basic statistics on the filtered dataset are summarized in Table 1.

**Table 1:** Some basic statistics about the dataset of films used for the analysis

	Number of single entities	Number of connections with movies
Movies	47,266	129,657
Genres	26	108,093
Countries	157	59,822
Directors	22,100	55,502
Actors	255,280	558,631
Actresses	143,771	265,954

For each entity, the number single occurrences and the number of connections it creates with movies are reported

**Fig. 1:**
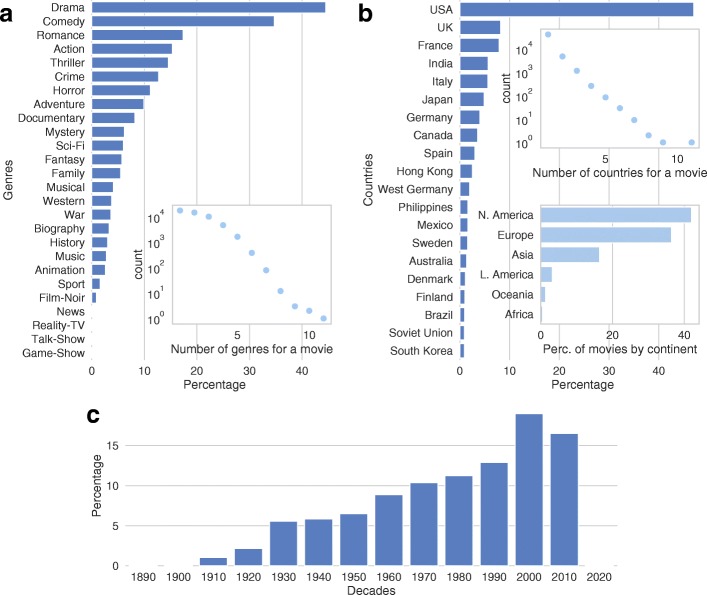
Percentage of movies by genre, country of production and year of first release. **a** Genres, **b** Countries, **c** Years of first release, grouped by decade

The distributions of years of first release (aggregated by decades), genres and countries of movies in dataset are reported in Fig. 1. We observe that the dataset contains data on releases in a range from the last years of Nineteenth century, when cinema has born, to the early years of 2020, as it also includes some movies already announced as in production, such as sequels of famous franchises. The number of films increases over the years (Fig. 1c), both because the industry of cinema has growth through the ages, and because users of IMDb could be more interested in adding information about brand new releases with respect to older ones. Many movies have been labeled with only one or two genres (Fig. 1a): the most employed one is “drama”, followed by “comedy”: with respect to our previous analysis in (Bioglio and Pensa 2018) the number of movies labeled with the latter has greatly reduced, suggesting that a huge number of “short” movies, removed from the dataset, were also labeled as “comedy”. Finally, around 90% of movies have been produced in only one country, and United States lead the ranking as the country with the largest film production over time (Fig. 1b): almost half of the movies in dataset have been produced there, while no one of the other countries is able to reach the 10% of entries. This result is probably affected by the fact that IMDb’s website is in English and is owned by an American company, but it leads to an important consideration: movies and references contained in the dataset are significant for an American audience. If we group the productivity by continent instead of country (Fig. 1c) we notice that also Europe can compete with United States in terms of movie production: then we can suppose that the dataset includes also movies significant for an European audience. Since the dataset is highly biased toward European and North American productions, we can consider the analysis contained in this paper as relevant principally for Western countries.

**Fig. 2:**
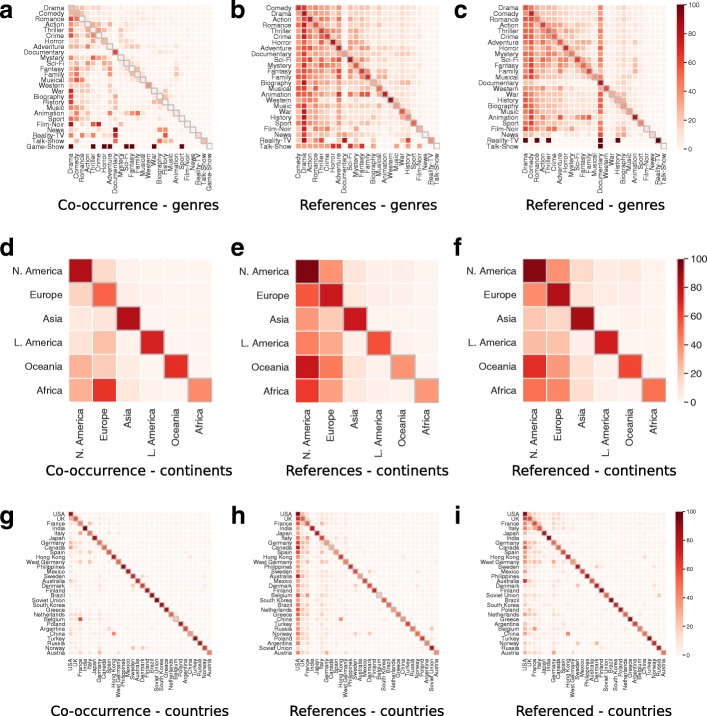
Densities of relationships between tags in Genres (**a**, **b**, **c**), Continents (**d**, **e**, **f**) and Countries (**g**, **h**, **i**): (**a**, **d**, **g**) shows how much a tag is used with other ones, divided by total number of movies belonging to the first tag, using the diagonal for counting when a tag is used alone; (**b**, **e**, **h**) show how much a tag references the other ones, divided by total number of movies belonging to the tag that makes at least one reference; (**c**, **f**, **g**) show how much a tag is referenced by the other ones, divided by total number of movies belonging to the tag that receives at least one reference. Since a movie can be labeled with multiple tags in the same item, rows sum to values bigger than one

We continue on analyzing the dataset by studying the relationships emerging between tags of the same item (country of production, year of release and genre): Fig. 2 graphically summarizes the relations found in genres and countries (also grouped by continents). It is worth noting that each movie can be labeled with more than one tag for a single item (for example, it can belong to multiple genres), then rows of heatmaps in Fig. 2 sum to values bigger than one. We start by analyzing the co-occurence between tags of the same item (Fig. 2a, d, g), that is the number of times the tag on *y*-axis and the one on *x*-axis label the same movie, divided by the total number of movies labeled with the tag on *y*-axis; values on diagonal represents the fraction of times the tag is the unique one for a movie. Co-occurrence of countries (Fig. 2g) is particularly relevant, because it counts the density of co-productions between two nations, and then we can consider it as a sort of index of collaborations between them. India, Soviet Union, Philippines, United States and Japan tend to produce movies without the collaboration of other countries, while other nations seem less independent: we can observe strong collaborations between European countries, in particular between Belgium and France (probably due to the common language and frequent cultural exchanges, also noticeable in other arts, such as in music); between France, Spain and Italy; among countries in Scandinavia. A strong collaboration between China and Hong Kong emerges as well. When we broaden the look to continents (Fig. 2d), internal collaborations (on diagonal) still stand out, but it also emerges a significant collaboration between African and European countries.

As far as genres are concerned, “drama” movies are related to many tags that suggest a dramatic plot, such as “war”, “crime” and “thriller”, but also to some surprising ones: for example there are more romantic movies that are also labeled as dramatic than comedic, while most movies dealing with historical facts (the ones labeled as “biography” or “history”) describe dramatic events. On the other hand, the tags showing less co-occurrences with other ones are “documentary”, that presents (presumably) objective facts hardly connected with other tags more suited for fiction, and “western”, that probably exhibits peculiar features not in common with other genres.

As an example of different kind of relationship involving pairs of tags of the same category, we also study the references between them, i.e., the number of times a movie labeled with a tag, on the *y*-axis, references (resp. is referenced by) another movie with a tag on the *x*-axis, divided by the total number of movies labeled with the tag on the *y*-axis that makes (resp. receives) at least one reference. Such relationship is showed for genres in Fig. 2b (resp. Fig. 2c), for continents in Fig. 2e (resp. Fig. 2f), for countries in Fig. 2h (resp. Fig. 2i). When referred to countries, such value indicates a sort of cultural bias that one nation has w.r.t. the other one. Almost every country references films produced in United States: such observation shows the worldwide diffusion of American movies, as well as their cultural and economical dominance in the last century, but on the other hand is another partial evidence of the bias of data, that is even more visible at continental level (Fig. 2e). We also notice that the pairs of countries with a significant co-occurrence also exhibit a relationship in terms of references. When we analyze the countries that are referenced by the other ones we notice that Australian movies are quite influential for United States, and while India and Japan are influenced in the same way from United States, the latter are subject to a moderate influence from Japanese production, while Indian movies tends to be less influential outside their national borders. When we move our focus on genres, we observe that movies labeled as “drama”, “horror”, “sci-fi” and “western” mostly tend to reference other films of the same genre, while the genre that is less referenced by other ones is “documentary”; such behavior is even more highlighted if we look at the references received by other movies, where we can also notice that movies labeled as “animation”, “horror” and, quite surprisingly, “comedy” are principally referenced by films of the same genre.

## Ranking method

In this section we briefly introduce how our score for movies is calculated, and we justify our choice of the four centrality scores it considers.

We model networks as graphs *G*=(*V*,*E*), consisting in a set of nodes *V* and a set of edges *E* connecting the nodes. We consider only directed networks, where the set of edges *E* contains ordered pairs of nodes (*u*,*v*): *u*,*v*∈*V* representing a connection that goes from node *u* to *v*. *N*(*v*) denotes the set of in-neighbors of node *v*, more formally *N*(*v*)={*u*∈*V*:(*u*,*v*)∈*E*}, while *O*(*v*) denotes the set of out-neighbors of node *v*, that is *O*(*v*)={*u*∈*V*:(*v*,*u*)∈*E*}. A path between nodes *v*_0_ and *v*_*n*_ is defined as *P*(*v*_0_,*v*_*n*_)=*v*_0_,*v*_1_,...,*v*_*n*_, i.e. a sequence of nodes such that ∀*v*_*i*_∈*P*(*v*_0_,*v*_*n*_), *v*_*i*_∈*V* and (*v*_*i*_,*v*_*i*+1_)∈*E* for 0≤*i*<*n*. The distance *d*(*v*,*u*) between two nodes *v*,*u*∈*V* is calculated as the length of the shortest path from *v* to *u*; if *u* can not be reached from *v* the distance is *∞*.

As in Bioglio and Pensa (2018), we use IMDb data on references between films for calculating a score of each movie, based on how much it has been referenced by other movies released after its production: the idea is that a reference made to another movie is a sort of clue left by some member of the crew, suggesting that the referenced film has influenced him or her in some way. In order to calculate our ranking, we construct a references network *G*=(*V*,*E*), where nodes *V* are films, and there exists a directed edge (*u*,*v*)∈*E* between two nodes *u* and *v* if the first one makes a reference to the second one.

The score of a movie is calculated by combining four different centrality scores: in-degree, closeness, harmonic and PageRank centrality. The in-degree centrality of a given node *v* is simply the number of incoming edges:

$$I(v) = N(v)$$

Closeness centrality (Bavelas 1950) is calculated as the sum of the length of the shortest paths between a given node and all other nodes in the graph:

$$C(v) = \frac{1}{\sum_{u \in V,\, d(u,v)\neq\infty} d(u, v)}$$

Harmonic centrality (Dekker 2005; Rochat 2009) for a node *v* is the sum of the reciprocal of shortest path distances from all other nodes to *v*, more formally:

$$H(v) = \sum_{u \in V,\, d(u,v)\neq\infty}\frac{1}{d(u, v)}$$

Finally, PageRank centrality (Brin and Page 1998) is based on left dominant eigenvector, counting the number of possible ways any other node can reach the node under study. It is a well-known measure because is one of the many factors used by Google search engine to determine the ranking of web pages as they appear in a search result. For a given node *v* its PageRank score is defined as:

$$P(v)=d\boldsymbol{a}_{v} P(v) + \frac{(1-d)}{n}$$

where *d*=[0,1] is the damping factor (the 1−*d* quantity is also known as restart probability), and ***a***_*v*_=[*a*_1*v*_,…,*a*_*nv*_] is a vector such that each element *a*_*iv*_=1/|*O*(*u*)| if there exists a directed edge (*v*_*i*_,*v*) (*a*_*iv*_=0 otherwise). For more details on centrality and centrality scores in graphs see (Boldi and Vigna 2014).

Each one of these centrality scores is calculated singularly, and normalized, obtaining a value between 0 and 1: our score is the weighted sum of these scores, where each one has the same weight of 0.25.

The calculation of centrality scores has been performed by means of Python scripts^5^, using the networkx library^6^ (Hagberg et al. 2008).

### Motivations

For the composition of our score, we have selected in-degree, closeness, harmonic and PageRank centralities because they best model our idea of importance for a movie in terms of influence, letting us to detect the pieces that had been an inspiring role in cinema. In-degree centrality takes into account the number of references received by a movie: it can be considered as the simplest measure of influence, because a movie that has played a relevant role in cinema should be highly referenced in the field, as happens for scientific publications. Both closeness and harmonic centralities quantify, in a slightly different way, the distance from a node to all the other ones: movies with high score on these measures are highly referenced by movies that are, in turn, highly referenced. For this reason these measures give an additional information with respect to in-degree centrality, detecting films that inspire other important ones, and for this reason have had an high influential role in the history of cinema. PageRank goes a step further: it counts a weighted number of references received by a movie, where the weight of a link is related to the number of references received by the incoming node. In this way, it rewards movies that are referenced by important (in terms of references received) movies. Summarizing, each of these scores evaluates a different concept of centrality, and each one is important for detecting influential movies: for this reason, we decided to use in our score a combination of all of them, weighted in the same way.

**Fig. 3:**
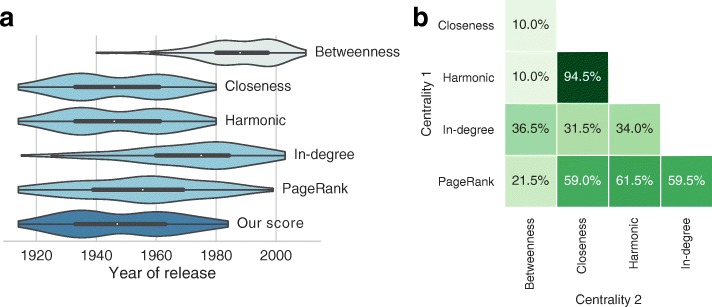
Statistics on centrality scores applied to our dataset. **a** Year of release of top 200 films according to different ranking methods, **b** Percentage of top 200 movies in common between two ranking methods

On the other hand, we decided to exclude from our method the path-based scores, like betweenness centrality, because they measure influence of nodes in terms of communication: a node with high betweenness score has an important role over information passing between other nodes, and its removal can disrupt communication paths inside the network, because it quantifies the number of times a node acts as a bridge along the shortest path between two other nodes. In this case, betweenness centrality can identify movies that have had an important role in diffusing ideas and trends, but in our score we are not interested in this kind of dynamics. In addition, such measure would reward movies released in intermediate ages, since they are likely to have both many movies to reference and to be referenced by. As a partial evidence of this behavior, Fig. 3a shows the year of release of the top 200 movies according to our score, to the four centrality scores we use and to betweenness centrality: it highlights a high bias of the latter score toward movies released after the 80’s, while the other ones reward films released between the 40’s and the 70’s, with unique distributions (except for closeness and harmonic centralities, that show very similar distributions). In addition, Fig. 3b reports the percentage of movies in common between the top 200 films as determined by the two centrality measures: we observe that betweenness shows a very low relationship with the other scores, because of its bias to more modern movies, with the sole exception of in-degree. From the same figure we can also notice that closeness and harmonic measures lead to very similar lists of top movies, but there still exist some differences between them. Top ranked movies according to betweenness could surely be interesting, but they are far from meeting the objective of our study, whose aim is to focus on milestones pieces in terms of influence and inspiration, then we decided not to include it, as well as other path-based measures, in our score.

## Results of ranking

In this section, we present and comment the results of our ranking method applied to two networks of references: the one among movies described in “Analysis of the dataset” section, and the one among directors, obtained by joining the network in “Analysis of the dataset” section with data about film directions collected from IMDb.

### Ranking of movies

Even after excluding two genres (“adult” and “short”, as described in “Analysis of the dataset” section) from the dataset, the top 20 movies, reported in Table 2, are the same previously discovered in (Bioglio and Pensa 2018), even if some title is in a slight different position. As expected from the distribution of movies according to their country of production in Fig. 1b, most movies in the list have been produced in the United States, and the few produced outside such country have been released in the early years of cinema. Interestingly, all the movies in this list have been released before the 80’s, and most of them even before the 40’s: such a result is not completely surprising, because our score measures the influence of movies in history, then classical films, representing the first steps and experimentations in cinematic arts, have more probability of having influenced the following ones.

**Table 2:** Top 20 movies by influence centrality

Rank	Title	Rank	Title
1	The Wizard of Oz (1939)	11	Casablanca (1942)
2	Star Wars (1977)	12	Dracula (1931)
3	Psycho (1960)	13	The Godfather (1972)
4	King Kong (1933)	14	Jaws (1975)
5	2001: A Space Odyssey (1968)	15	Nosferatu, eine Symphonie... (1922)
6	Metropolis (1927)	16	The Searchers (1956)
7	Citizen Kane (1941)	17	Cabiria (1914)
8	The Birth of a Nation (1915)	18	Dr. Strangelove or: How I...(1964)
9	Frankenstein (1931)	19	Gone with the Wind (1939)
10	Snow White and the Seven Dwarfs (1937)	20	Bronenosets Potemkin (1925)

**Fig. 4:**
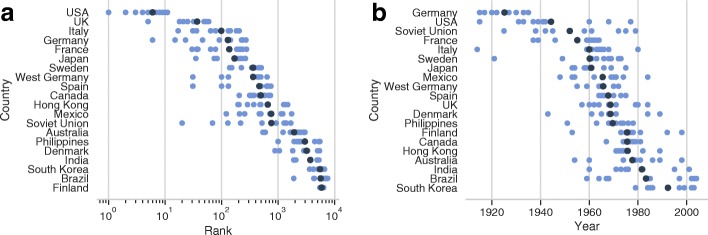
Rank and year of release of top 10 movies in each country, in cyan: dark points represent average. **a** Rank, **b** Year of release

If we analyze the performances of single countries, focusing on the average ranking of top 10 movies produced in each nation (Fig. 4a), the United States lead the rank, followed by several European and North American countries: the only non-Western nation in this list is Japan. If we look at the year of release of top 10 movies produced in each country, we observe two kinds of pattern: some countries have produced influential movies for a widespread period of time, like the United States, the Soviet Union, the United Kingdom and Australia, while other ones exhibit a clear peak during a small range of years, like Germany, Italy, Sweden and Hong Kong. A curious exception is France, that shows two peaks of influence, in the 40’s and 60’s. It is also worth noting that Asian movies, with the only exception of Japanese ones, tends to become relevant later than those produced in Western countries, and their global rank is low.

To validate our findings, we select the top 25 movies produced in the United States according to our ranking, and we count how many of them belong to two lists of significant movies established by experts. We found that 84% of movies belong to the United States Library of Congress’s National Film Registry^7^ (NFR), that contains 703 movies produced in the United States that are “culturally, historically, or aesthetically significant”, while 76% of them is included in the 122 best American movies determined by the American Film Institute (AFI).^8^

### Ranking of directors

In addition to our previous analysis in Bioglio and Pensa (2018), here we also employ the references between movies for calculating an inference score for directors, by constructing a network of references among them. The resulting graph contains directors as nodes, and an oriented edge between two nodes every time the first director has referenced, in a movie directed by her or him, some other movie directed by the second one. This operation (extending the network of references to directors) is semantically meaningful, because directors are the ones in charge of controlling artistic and dramatic aspects of films, they visualize the screenplay and guide both technical crew and actors during the shots, and usually they also have a key role in choosing cast members, production design, and other creative aspects of filmmaking. Due to their central role in the production of movies, it is probable that any reference to other movies contained in their films have been decided, or at least approved, by them, and then represents their wish and choice of referencing the work of a colleague. We then apply the same algorithm used for the network of movies to compute a ranking between directors: the results for the top 20 directors obtained with this method is listed on Table 3.

**Table 3:** Top 20 directors by influence centrality, with information on range of their career

Rank	Name	Career	Rank	Name	Career
1	Cukor, George	(1930 - 1981)	11	Whale, James (I)	(1930 - 1940)
2	Fleming, Victor (I)	(1920 - 1948)	12	Coppola, Francis Ford	(1959 - 2011)
3	Hitchcock, Alfred (I)	(1925 - 1976)	13	Cameron, James (I)	(1981 - 2025)
4	LeRoy, Mervyn	(1928 - 1968)	14	Jackson, Wilfred (I)	(1937 - 1955)
5	Spielberg, Steven	(1964 - 2020)	15	Wood, Sam (I)	(1920 - 1949)
6	Kubrick, Stanley	(1953 - 1999)	16	Schoedsack, Ernest B.	(1925 - 1952)
7	Taurog, Norman	(1930 - 1968)	17	Cooper, Merian C.	(1925 - 1952)
8	Vidor, King	(1921 - 1959)	18	Wilder, Billy	(1942 - 1981)
9	Lucas, George (I)	(1969 - 2005)	19	Welles, Orson	(1938 - 1993)
10	Curtiz, Michael	(1922 - 1961)	20	Hawks, Howard	(1927 - 1970)

Most of the directors in the top part of the ranking have directed some movie belonging to the top 20 movies in Table 2. In particular all the five persons that IMDb credits as directors of “The wizard of Oz”, the top movie in our list, belong to the top 8 list: the relative position of each one depends on the influence of other movies directed by him. George Cukor and Victor Fleming lead the ranking probably because of their participation to “Gone with the wind”, the movie in the 19th position of our movie ranking (and Sam Wood, its third director, is ranked 15th): the former gains the top probably thanks to its role in other very influential movies, such as “My Fair Lady” and “The Philadelphia Story”. The only directors that belong to the top 8 ranking without having directed “The wizard of Oz” are Hitchcock, Spielberg and Kubrick, but all of them have directed at least one of the 20 most influential movies. After them, we find the directors of “Star Wars”, “Casablanca”, “Frankenstein”, and “The Godfather”, followed by the first person that has not directed any of the movies in the top 20 list, James Cameron: it is really impressive that he was able to reach this position even if his career starts only after the 80’s, much later than the other ones in the list. Even more surprisingly, he has directed only a dozen of movies, but among them there are some of the most influential films of modern times, such as “The Terminator” series, “Titanic” and “Avatar”. The other directors in the list without a movie belonging to the top 20 ranking are Billy Wilder and Howard Hawks.

The list in Table 3 gives an idea of the influence of directors in the whole history of cinema, but it compares persons who have worked in different ages, and involved in different genres. It should be interesting to analyze the ranking of directors in analogous classes, as done for movies in Bioglio and Pensa (2018), but in the case of directors it is more difficult to identify a clear way for labeling them: a director during her career works on several movies, usually belonging to different genres (even if some directors prefer to focus their career on a small subset of genres, for example “horror” movies) and produced in different countries (for example several Asian and European directors begin their career in native country and then move to the United States after having gained international reputation). For this reason, we decide to use a different method for analyzing the career of directors, that is described in “Analysis of careers of directors, actors and actresses” section: since such method is not strongly related to directors, we decide to extend it also to actors and actresses.

## Analysis of careers of directors, actors and actresses

The method employed in “Results of ranking” section for directors is not applicable to other members of the crew, because none of them has (usually) an influence and a decisional power comparable to director during the production of a movie. A different way of considering the career of a person, compared to the one used for directors in “Results of ranking” section, is to evaluate the importance of movies he took part. However, in our case, influence is calculated as centrality in the network of references, then older movies tends to achieve a better ranking than new ones, and their crew would benefit from this circumstance. For avoiding the effect of time, and then comparing influence of movies with same ages, we adopt a ranking similar to the Medal Ranking System employed for the Olympic Games. We assign our score to each movie, according to the method described in “Results of ranking” section, then, in each year, we select the films in the top 5% ranking, assigning to each member of its crew a “gold” point; we repeat the algorithm for movies from the top 5% to 10% ranking, assigning a “silver” point, and for movies from the top 10% to 15%, whose crew members gain a “bronze” point. The ranking of persons is ordered by looking to the number of “gold” points gained, then “silver” and finally “bronze” ones. Hence, we take into account the participation of a person in the most influential movies released in a year, and her ranking is due to the number of top movies she took part during her career. In the event of all points being equal, the individuals are ranked according to the overall number of movies they contribute to, in ascending order, since we prefer to reward who was capable to reach the same level collaborating in less movies. In addition, this method allow us to filter members of the crew according to their involvement in a movie labeled with a specific tag, obtaining the most important personalities according to each tag. We limit our research to movies released before 2010: we estimate, in fact, that it is difficult for a film to gain a realistic (and measurable) influence in less than 10 years.

### Directors

Table 4 shows the ranking of the top 20 directors of all times according to our method. Contrary to the ranking of most influential directors on Table 3, here we notice a greater mixture of ages (careers range from early years of the 20th century to early years of the 21th century), nationalities (directors hailing from the United States and the United Kingdom are still the majority, but there is significative presence of Swedish, Japanese and Austrian directors), and number of movies directed (from around a dozen to almost 80). Such heterogeneity is exactly the objective of this method, while the one based only on influence is biased towards older movies rather than modern ones. The homogeneity of the previous ranking highlights the influence of ancient masterpieces at the price of dimming most recent works.

**Table 4:** Top 20 directors: each item contains the number of golden (G), silver (S) and Bronze (B) points collected, the year of the first and last movies, and the number of films directed in total

Rank	Name	G	S	B	Career	Movies
1	Hitchcock, Alfred (I)	22	9	4	(1925 - 1976)	47
2	Spielberg, Steven	18	3	2	(1964 - 2020)	33
3	De Palma, Brian	14	5	0	(1968 - 2012)	29
4	Hawks, Howard	12	4	3	(1927 - 1970)	39
5	Ford, John (I)	12	4	2	(1917 - 1966)	59
6	Scorsese, Martin (I)	11	6	2	(1967 - 2014)	33
7	Bergman, Ingmar	11	2	1	(1946 - 1986)	33
8	Kubrick, Stanley	11	1	1	(1953 - 1999)	13
9	Thomas, Gerald (I)	11	1	1	(1958 - 1992)	33
10	Honda, Ishirō	10	8	2	(1954 - 1990)	30
11	Lang, Fritz (I)	10	1	2	(1919 - 1960)	33
12	Tarantino, Quentin	10	1	0	(1987 - 2015)	13
13	Cronenberg, David	10	1	0	(1975 - 2014)	19
14	Curtiz, Michael	9	12	7	(1922 - 1961)	77
15	Walsh, Raoul	9	5	4	(1914 - 1964)	57
16	Carpenter, John (I)	9	5	2	(1974 - 2010)	18
17	Eastwood, Clint	9	4	4	(1971 - 2016)	34
18	Burton, Tim (I)	9	2	2	(1985 - 2019)	17
19	Landis, John (I)	9	2	1	(1973 - 2010)	19
20	Rodriguez, Robert (I)	9	2	0	(1992 - 2014)	18

The list in Table 4 shows directors with the most impressive careers in the entire history of cinema, but there are personalities that have been very important only for few years, or in certain genres, then we decided to extend the research also on specific tags: we apply the same “medal” method, but filtering only the participation in movies labeled with the tag under study. We start by analyzing the production of directors in each decade, summarizing our findings in Table 5.

**Table 5:** Top 5 directors by decade, from 40’s to 90’s

Rank	Name	Points	Name	Points	Name	Points
	1940-49		1950-59		1960-69	
1	Hitchcock, Alfred (I)	7, 3, 1	Hitchcock, Alfred (I)	8, 1, 1	Thomas, Gerald (I)	8, 1, 1
2	Walsh, Raoul	6, 1, 2	Honda, Ishirō	6, 0, 1	Godard, Jean-Luc	6, 0, 5
3	Welles, Orson	5, 0, 1	Fisher, Terence	4, 2, 0	Bergman, Ingmar	6, 0, 0
4	Ford, John (I)	5, 0, 0	Arnold, Jack (I)	4, 1, 1	Corbucci, Sergio	5, 1, 0
5	Hawks, Howard	4, 3, 0	Hawks, Howard	4, 0, 0	Honda, Ishirō	4, 6, 0
	1970-79		1980-89		1990-99	
1	Altman, Robert (I)	5, 1, 1	Carpenter, John (I)	6, 1, 1	Spielberg, Steven	5, 1, 0
2	De Palma, Brian	5, 0, 0	Landis, John (I)	6, 1, 0	Burton, Tim (I)	5, 0, 0
3	Eastwood, Clint	4, 1, 0	De Palma, Brian	5, 1, 0	Coen, Joel & Ethan	5, 0, 0
4	Coppola, Francis Ford	4, 0, 0	Spielberg, Steven	5, 0, 2	Fincher, David	4, 0, 0
5	Lumet, Sidney	3, 1, 2	Fulci, Lucio	4, 3, 0	Sonnenfeld, Barry	3, 2, 1

For each individual it is reported the number of movies she has directed during the decade that reached top 5%, 10% and 15% influence in their year of release

By analyzing the production of each director during ten years only, we observe that results are more homogeneous: the director that reaches the top ranking in each list has from 5 to 8 movies in top 5%, while the ones in 5th position has directed around 3 movies that are most influential according to our method. It is also interesting that no one filmmaker in the list of 60’s comes from United States, contrary to other ages, where American directors are the majority: such results shows how much European and Asian cinema of that period has been influential for the following productions.

We perform a similar analysis on genres, and display in Table 6 the top 5 directors in four peculiar genres: “comedy”, “horror”, “crime” and “western”; these genres have been selected because each of them has its distinctive aesthetic and rules, significantly dissimilar to other genres’ ones. As expected from distribution of genres in Fig. 1a, the “western” genre contains lower points than the other ones, because there are fewer movies labeled with this tag. We can also notice temporal patterns for each genre: “western” has had a great impact in the last years of the 30’s and in the middle of 60’s, “crime” has emerged after the 70’s (with another peak at the end of 80’s), while “horror” seems to emerge in the 60’s and to reach its peak of influence during 70’s; on the other hand, “comedy” is widespread during the ages. For “western”, we can also notice that two directors in the list belong to the sub-genre called ‘Spaghetti Western’, that emerged in Italy during the mid-60s, showing the importance of this style for the genre.

**Table 6:** Top 5 directors by genre, for comedy, horror, crime and western movies

Rank	Name	Points	Years	Name	Points	Years
	Comedy			Horror		
1	Thomas, Gerald (I)	11, 1, 1	’58-’69	Craven, Wes	9, 1, 1	’72-’00
2	Landis, John (I)	8, 2, 1	’73-’98	Cronenberg, David	8, 1, 0	’75-’99
3	Waters, John (I)	6, 4, 2	’69-’04	Fisher, Terence	6, 3, 2	’57-’69
4	Brooks, Mel	5, 4, 1	’67-’93	Carpenter, John (I)	6, 2, 2	’78-’01
5	McCarey, Leo	5, 0, 1	’30-’44	Fulci, Lucio	6, 1, 0	’72-’83
	Crime			Western		
1	Scorsese, Martin (I)	8, 1, 1	’72-’06	Ford, John (I)	7, 2, 1	’24-’64
2	Coen, Joel & Ethan	6, 2, 2	’84-’08	Corbucci, Sergio	6, 0, 0	’66-’70
3	Woo, John (I)	6, 0, 1	’86-’97	Selander, Lesley	5, 5, 0	’38-’41
4	Tarantino, Quentin	6, 0, 0	’92-’05	Leone, Sergio (I)	4, 0, 0	’64-’68
5	De Palma, Brian	5, 2, 0	’68-’06	Eastwood, Clint	3, 1, 0	’73-’92

For each individual, we report the number of movies he has directed during the decade that reached top 5%, 10% and 15% influence in their year of release, and their range of release years

Finally, we also filter the results by country, limiting our study to the four countries having more movies in the dataset, as depicted in Fig. 1b, i.e., the United States, the United Kingdom, France and Italy: results are summarized in Table 7. India has been excluded because, as showed in Figs. 2i and 4a, its influence on other countries is too weak, then the identities of Indian filmmakers are relatively unknown outside their native country. It is not surprising that the points reached by directors from the United States almost double the ones of other countries, since the number of movies produced there is several times greater than outside, as showed in Fig. 1b. Additionally, the influence of their directors ranges over all the ages of cinema, from the very beginning to recent times; something similar happens for directors that worked in the United Kingdom. The case of Alfred Hitchcock is particularly interesting: after reaching celebrity in the United Kingdom, he moves to the United States, continuing to reap success. As regards the other two countries under study, we notice that French directors have been influential during the 60’s, in the period called ‘French New Wave’ (*La Nouvelle Vague*), while Italian ones during the 70’s. In addition, the presence of the same filmmaker, Federico Fellini, in the lists of both countries suggests the existence of a strong collaboration between them, as already noticed in Fig. 2g.

**Table 7:** Top 5 directors by country, for movies produced in the United States, the United Kingdom, France and Italy

Rank	Name	Points	Years	Name	Points	Years
	United States			United Kingdom		
1	Hitchcock, Alfred (I)	19, 3, 3	’40-’76	Thomas, Gerald (I)	11, 1, 1	’58-’69
2	Spielberg, Steven	18, 3, 2	’64-’08	Kubrick, Stanley	7, 1, 0	’62-’99
3	De Palma, Brian	14, 5, 0	’68-’06	Scott, Ridley	7, 0, 2	’77-’07
4	Hawks, Howard	12, 4, 3	’30-’67	Fisher, Terence	6, 4, 2	’50-’69
5	Ford, John (I)	12, 4, 2	’24-’64	Hitchcock, Alfred (I)	5, 6, 1	’27-’72
	France			Italy		
1	Godard, Jean-Luc	6, 0, 5	’60-’67	Fulci, Lucio	6, 5, 0	’71-’84
2	Truffaut, François	4, 1 0	’59-’72	Fellini, Federico	6, 2, 4	’52-’83
3	Renoir, Jean	4, 0, 1	’32-’39	Corbucci, Sergio	6, 1, 0	’64-’70
4	Fellini, Federico	3, 1, 4	’53-’83	Argento, Dario	5, 5, 0	’70-’96
5	Melville, Jean-Pierre	3, 1, 1	’56-’70	Bava, Mario	5, 4, 3	’60-’75

For each individual, we report the number of movies he has directed, and that has been produced in the country, that reached top 5%, 10% and 15% influence in their year of release, and their range of release years

### Actors and actresses

Table 8 provides the ranking of actors and actresses according to our method: as done with directors, the analysis is limited to movies released before 2010, even if, for columns “career” and “number of movies”, we have also taken into account films released after such year. Looking at the list, there are several interesting results. First, all personalities come from the United States or the United Kingdom, showing both the great impact that movies in English have in the industry of cinema, and the bias of the dataset. As second point, the great majority of individuals are still in activity or, at least, appeared in a movie released after 2010, suggesting that the most influential movies of modern times are the ones starring most famous actors and actresses. Probably such result is due to little bias that affect modern movies released after 90’s: since they are too recent for having already deeply influenced other ones, their score is heavily influenced by the presence of sequel or other movies belonging to the same franchise. This kind of films usually have a big budget, that can be spent for hiring famous actors and actresses, and attract more audience. The same happened also in the past, but movies that have been popular during their release usually may have a great impact on the contemporary ones, although their importance may weaken for films released many years later. This result is also probably affected by the increase in production of movies over the years observed in Fig. 1c: since our method select a percentage of movies released each year, if the number of released films grows, the same happens to the number of movies it selects. We could select a fixed number of movies instead of a certain percentage, but in this case we would foster films released in less productive years, to the detriment of more prolific ages. Finally, and most crucially, the number of top movies starring a male actor is several way greater than the number of top movies starring an actress: among them, only Lois Maxwell, who appeared in most movies of James Bond film series with the role of M’s secretary, can compete with her male colleagues, entering in the bottom part of the top ten ranking. This result can be partially justified by the differences in number of actors and actresses in our dataset, reported in Table 1, with the former almost doubling the second. The same happens to the number of connections with movies: however the list shows only top personalities, that in case of gender equality should be comparable.

**Table 8:** Top 20 actors and actresses: each item contains the number of golden (G), silver (S) and Bronze (B) points collected, the year of the first and last movies, and the number of films starred in total

Rank	Name	Points	Career	Mov.	Name	Points	Career	Mov.
	Actors				Actresses			
1	Jackson, Samuel L.	24, 5, 6	’81-’17	82	Maxwell, Lois	16, 2, 0	’47-’88	27
2	Eastwood, Clint	18, 6, 4	’55-’13	54	Fisher, Carrie (I)	11, 3, 0	’75-’15	34
3	Cruise, Tom	18, 4, 3	’81-’17	41	O’Sullivan, M. (I)	11, 2, 2	’30-’86	43
4	Schwarzenegger, A.	18, 3, 3	’76-’15	38	Berry, Halle	10, 2, 4	’91-’17	29
5	Wayne, John (I)	16, 10, 9	’30-’76	112	Barrymore, D. (I)	9, 6, 3	’80-’15	45
6	Dafoe, Willem	16, 7, 5	’83-’14	57	Shaye, Lin	9, 5, 6	’78-’16	61
7	Willis, Bruce	16, 6, 3	’87-’16	62	Diaz, Cameron	9, 2, 2	’94-’14	29
8	Price, Vincent (I)	16, 5, 4	’38-’91	75	Moore, Julianne	8, 5, 4	’90-’17	49
9	Llewelyn, Desmond	16, 2, 0	’63-’99	18	Dunaway, Faye	8, 5, 3	’67-’07	41
10	Bond, Ward	16, 1, 4	’29-’59	73	Grant, Beth (I)	8, 4, 3	’87-’15	41
11	De Niro, Robert	15, 13, 7	’68-’16	75	Curtis, Jamie Lee	8, 4, 3	’78-’14	33
13	Connery, Sean	15, 8, 10	’57-’03	52	Christie, Julie (I)	8, 4, 2	’63-’12	27
12	Nicholson, Jack (I)	15, 8, 4	’58-’11	54	Weaver, Sigourney	8, 3, 5	’77-’16	46
14	Ford, Harrison (I)	15, 6, 3	’68-’15	45	Crawford, Joan (I)	8, 3, 4	’25-’70	57
15	Trejo, Danny	15, 3, 3	’87-’15	74	Smith, Maggie (I)	8, 3, 3	’62-’15	43
16	Lee, Christopher (I)	14, 15, 7	’48-’14	105	Bay, Frances	8, 3, 1	’78-’14	35
17	Coltrane, Robbie	14, 3, 0	’80-’12	37	Trainor, Mary Ellen	8, 2, 6	’84-’03	23
18	Depp, Johnny	14, 2, 4	’84-’17	52	Leachman, Cloris	8, 2, 4	’55-’16	38
19	Buscemi, Steve (I)	13, 7, 4	’86-’16	70	Dench, Judi	8, 2, 1	’65-’16	33
20	Stewart, James (I)	13, 6, 4	’35-’91	64	Portman, Natalie	8, 1, 1	’94-’17	28

As done for directors, we also study the top ranking filtering by decade: results for both actors and actresses in decades from the 40’s to the 90’s are reported in Table 9: we notice that the amount of points for actors and actresses through ages is more homogeneous, with the exception of the 90’s, which registers a higher value of movies in the top influence: this observation strengthen the hypothesis of the small bias that affects modern movies. It is curious to notice that the ranking is again almost entirely composed by American personalities, except for male actors in the 50’s, dominated by Japan: some of them had a role in the works by Akira Kurosawa, a very influential Japanese director, while some others acted in several Kaiju (Japanese monsters) movies, like the ones of Godzilla franchise, that have had a great influence on American monster movies. However, a difference between male actors and actresses is still visible: among the latter, only Lois Maxwell in the 60’s (and maybe Jamie Lee Curtis in 80’s) can be included in the top 5 of best actors of any gender.

**Table 9:** Top 5 actors and actresses by decade, from the 40’s to the 90’s

Rank	Name	Points	Name	Points	Name	Points
	1940-49		1950-59		1960-69	
1	Bond, Ward	9, 1, 2	Shimura, Takashi	7, 2, 2	Hawtrey, Charles (I)	7, 2, 1
2	Weissmuller, Johnny	8, 0, 2	Tsuchiya, Yoshio (I)	7, 0, 1	Williams, Kenneth (I)	7, 1, 1
3	Wayne, John (I)	7, 0, 2	Cushing, Peter	6, 1, 0	James, Sidney (II)	6, 1, 1
4	Cotten, Joseph (I)	6, 3, 2	Nakajima, Haruo	6, 0, 1	Lee, Bernard (I)	6, 1, 0
5	Trowbridge, C. (I)	6, 0, 1	Ōtomo, Shin	6, 0, 0	Wayne, John (I)	5, 5, 3
1	Joyce, Brenda (I)	5, 0, 0	Monroe, Marilyn	5, 2, 4	Maxwell, Lois	7, 1, 0
2	McShane, Kitty	4, 3, 1	Jones, Carolyn (I)	5, 0, 1	Taylor, Elizabeth (I)	5, 0, 1
3	Bergman, Ingrid (I)	4, 2, 2	Grahame, Gloria	4, 3, 0	Sims, Joan (I)	4, 1, 1
4	Hayworth, Rita	4, 1, 2	Kelly, Grace (I)	4, 1, 1	Houston, Renee (I)	4, 0, 1
5	Anderson, Judith (I)	4, 0, 2	Jacques, Hattie	4, 0, 1	Thulin, Ingrid	4, 0, 0
	1970-79		1980-89		1990-99	
1	Eastwood, Clint	8, 4, 0	Schwarzenegger, A.	8, 0, 0	Jackson, Samuel L.	12, 3, 5
2	Beatty, Ned	8, 1, 2	Aykroyd, Dan	7, 2, 0	Willis, Bruce	10, 4, 2
3	Duvall, Robert	6, 2, 2	Miller, Dick (I)	6, 3, 3	Buscemi, Steve (I)	10, 3, 3
4	Sutherland, D. (I)	6, 1, 4	Ford, Harrison (I)	6, 1, 1	Sizemore, Tom (I)	8, 3, 1
5	James, Clifton (I)	6, 0, 1	Feldman, Corey (I)	6, 1, 0	Cruise, Tom	8, 0, 1
1	Maxwell, Lois	5, 0, 0	Curtis, Jamie Lee	6, 1, 0	Moore, Julianne	6, 2, 3
2	Leachman, Cloris	4, 2, 1	Trainor, Mary Ellen	5, 2, 0	Russo, Rene (I)	5, 4, 0
3	Christie, Julie (I)	4, 2, 0	Fisher, Carrie (I)	5, 1, 0	Ricci, Christina (I)	5, 3, 1
4	Dunaway, Faye	4, 2, 0	Shaye, Lin	4, 1, 2	Graham, Heather (I)	5, 2, 1
5	Shire, Talia	4, 0, 0	Barkin, Ellen	4, 0, 1	Walters, Melora	5, 1, 0

For each individual we report the number of movies he has directed during the decade that reached the top 5%, 10% and 15% influence in their year of release

We can find the same behavior if we filter careers by genre: the results for “comedy”, “horror”, “sci-fi” and “musical” movies are summarized in Table 10, where actresses are highlighted in bold. We report data on “musical” films because it is the only case that shows some gender equality: this kind of movies is characterized by many singing and dancing scenes, that are probably performed by male and female actors in equal rate because of producers’ decisions. It is also interesting to notice that no one of the actors in the “comedy” list belongs to the general ranking in Table 8, probably because dramatic movies are more influential. Furthermore, top 4 ranking in the “sci-fi” list is composed by Japanese actors, starting their career in the mid-50’s: as in the list by decades, all of them were involved in Japanese monster movies, that had a great influence on American cinema. As a final remark, all the actors in the “musical” list acted from the 30’s to the 40’s, showing that such genre of movies was very popular in that period, while it lost appeal in more recent times.

**Table 10:** Top 5 actors (of any gender, actresses in bold) by genre, for comedy, horror, sci-fi and musical movies

Rank	Name	Level	Years	Name	Level	Years
	Comedy			Horror		
1	Murray, Bill (I)	10, 4, 5	’80-’09	Price, Vincent (I)	9, 4, 2	’40-’74
2	Stiller, Ben	10, 3, 4	’96-’09	Lugosi, Bela	8, 4, 4	’31-’55
3	Hawtrey, Charles (I)	10, 3, 1	’42-’69	Savini, Tom	8, 0, 2	’78-’07
4	Carrey, Jim	10, 1, 3	’83-’04	Lee, Christopher (I)	6, 10, 5	’57-’99
5	Williams, Kenneth (I)	10, 1, 1	’58-’69	Karloff, Boris (I)	6, 7, 1	’32-’68
	Sci-fi			Musical		
1	Sahara, Kenji	7, 9, 1	’57-’04	**Garland, Judy**	4, 1, 3	’37-’54
2	Nakajima, Haruo	7, 7, 1	’54-’72	Kelly, Gene (I)	4, 0, 2	’44-’54
3	Tajima, Yoshifumi	7, 6, 1	’56-’84	**MacDonald, Jeanette (I)**	4, 0, 1	’29-’37
4	Tezuka, Katsumi	7, 3, 1	’54-’64	Astaire, Fred	3, 7, 6	’33-’57
5	Schwarzenegger, Arnold	7, 1, 1	’84-’03	**Dumont, Margaret**	3, 4, 0	’29-’41

For each individual we report the number of movies he has starred during the decade that reached the top 5%, 10% and 15% influence in their year of release, and their range of release years

Actresses’ names are emphasized in boldface

Finally, we analyze if such difference in gender only happens in movies produced in the United States and the United Kingdom, by filtering actors and actresses’ careers by country of production of the movies they are involved in. We notice that such behavior is a bit weaker in other European countries, as showed in Table 11 (actresses are reported in bold), but it is still present, with only one exception. In France and Italy the majority of top positions are occupied by male actors, but there is at least one actress in the last position, and their values can be compared with the other members in their list. The same happens in almost all other countries, with the exception of Sweden, where actresses are the majority, even if we extend the analysis to top 10 positions; if we analyze their career, we notice that all Swedish actors in list owe their influence to Ingmar Bergman, one of the most influential European directors, as also stated in Table 4. As additional country in Table 11 we report Japan, because it is the only one where actors have scores comparable with English movies, thanks to their participation in Monster movies, as we have already mentioned beforehand.

**Table 11:** Top 5 actors (of any gender, actresses in bold) by country, for movies produced in France, Italy, Sweden and Japan. For each individual we report the number of movies he has starred in the country that reached top the 5%, 10% and 15% influence in their year of release, and their range of release years

Rank	Name	Level	Years	Name	Level	Years
	France			Italy		
1	Reno, Jean (I)	4, 2, 1	’85-’06	Mingozzi, Fulvio	5, 3, 0	’70-’85
2	Modot, Gaston	4, 1, 1	’30-’53	Nero, Franco	5, 1, 1	’66-’87
3	Oldman, Gary (I)	4, 1, 0	’91-’97	Robledo, Lorenzo	5, 1, 0	’65-’70
4	Rufus (I)	4, 0, 0	’76-’01	Trieste, Leopoldo	4, 2, 2	’52-’86
5	**Jovovich, Milla**	4, 0, 0	’97-’07	**Valli, Alida**	4, 1, 3	’58-’80
	Sweden			Japan		
1	Björnstrand, Gunnar	6, 1, 1	’53-’78	Shimura, Takashi	12, 6, 3	’43-’80
2	von Sydow, Max (I)	6, 0, 0	’57-’68	Tsuchiya, Yoshio (I)	11, 3, 1	’54-’91
3	**Thulin, Ingrid**	6, 0, 0	’57-’72	Nakajima, Haruo	9, 7, 1	’54-’72
4	**Lindblom, Gunnel**	6, 0, 0	’57-’09	Ōtomo, Shin	9, 3, 0	’54-’65
5	**Ullmann, Liv**	4, 0, 1	’66-’78	Tajima, Yoshifumi	8, 6, 2	’55-’84

Actresses’ names are emphasized in boldface

## Conclusions

In this paper we have employed the data available in the official repository of IMDb movie dataset for analyzing trends and patterns involving the production of movies, and for identifying the most important films and personalities (directors, actors and actresses) in the history of cinema. As initial analysis, we have combined some data on single movies, such as year of release, genres and countries of production, with data on references between films for analyzing patterns that emerge according to each of these features. We have observed collaboration patterns between countries, for example among France, Italy and Spain, between China and Hong Kong, and in Scandinavian countries, and reference relations between different genres.

Combining four centrality scores (in-degree, closeness, harmonic and PageRank) computed on the network of references among movies, we have calculated an influence score for each movie in dataset, obtaining a ranking of films based on references received. We have also employed the same method on the network of references between directors in order to obtain the most influential directors, but the results appear to be too much related to top movies, because most of directors on top positions are the ones that directed movies with higher influence.

Consequently, we have proposed a different metric for evaluating the career of directors, actors and actresses, inspired by the Medal Ranking System used in Olympic Games: a person gains a “gold” point for each movie directed/acted that reaches the top 5% influence in its year of release, a “silver” point for each movie in the best 5 to 10% ranking and a “bronze” point for each movie in the best 10 to 15% ranking, still in comparison with the other movies released during the same year. Personalities are then ranked according to the number of points gained, evaluating most precious metals more highly. Persons are also filtered by year, country and genre of the movies they participate in, with the goal of identifying the presence of trends and analyzing them. We found interesting patterns involving “horror” and “western” movies, and that movies on Japanese monster filmed during 50’s have been very influential for Western cinema. Finally, we have also observed some hint of gender inequality in the careers of actors and actresses, because the former leads almost every ranking to the detriment of the latter, with the only exceptions of “musical” movies — where results show a moderate gender equality — and of those produced in Sweden — where actresses overwhelm actors in global ranking.

## Abbreviations

AFI: American Film Institute, a nonprofit educational arts organization that preserves the legacy of America’s film heritage, awards film and television artists, and educates the next generation of storytellers and filmmakers in the Unites States
IMDb: Internet Movie Database, an online database (available at http://www.imdb.com) of information related to movies and TV series, featuring metadata such as year, country, genre, cast, production crew, budget, box office revenue, and other
NFR: the United States Library of Congress’s National Film Registry, the selection of films that deserve preservation made by the board of the United States Library of Congress

## Footnotes

1 an example of this calculation is performed by *Box Office Mojo*, available at http://www.boxofficemojo.com/alltime/adjusted.htm.

2 Metacritic is a website that aggregates reviews of media products, showing an average score for each item. It is available at http://www.metacritic.com/.

3 Statistics on IMDb are available at http://www.imdb.com/stats.

4 More details at https://www.imdb.com/interfaces.

5 Available as jupyter notebooks at https://github.com/bioglio/cinema.

6 Available at https://networkx.github.io/.

7 available at https://www.loc.gov/programs/national-film-preservation-board/film-registry/ complete-national-film-registry-listing/.

8 the list is obtained by merging two different rankings made by the institute: one released in 1998, available at http://www.afi.com/100years/movies.aspx, and another one released in 2007, available at http://www.afi.com/100years/movies10.aspx. It is interesting to notice that only 3 movies have the same rank in the two lists, suggesting that ranks made by human experts are highly influenced by trends, opinions and age of writing.
